# Gastric Residual Volume as a Candidate Brain–Gut Signal in Neurocritical Care: An Exploratory, Hypothesis-Generating Retrospective Cohort Study

**DOI:** 10.3390/nu18183103

**Published:** 2026-09-21

**Authors:** Geun-Hee Lee, Jeong-Hyun Park, Su-Min Kim, Joon-Ho Lee, Kyeong-Won Ryu, Jeong-Am Ryu

**Affiliations:** 1 Department of Critical Care Medicine, Samsung Medical Center, Sungkyunkwan University School of Medicine, 81 Irwon-ro, Gangnam-gu, Seoul 06351, Republic of Korea; geunhee.lee@samsung.com (G.-H.L.); jeonghyun93.park@samsung.com (J.-H.P.); sumin6.kim@samsung.com (S.-M.K.); jooooonho.lee@samsung.com (J.-H.L.); 2 Department of Dietetics, Samsung Medical Center, 81 Irwon-ro, Gangnam-gu, Seoul 06351, Republic of Korea; kw.yu@samsung.com; 3 Department of Neurosurgery, Samsung Medical Center, Sungkyunkwan University School of Medicine, 81 Irwon-ro, Gangnam-gu, Seoul 06351, Republic of Korea

**Keywords:** gastric residual volume, intracranial pressure, neurocritical care, Glasgow Outcome Scale, enteral nutrition, brain–gut axis

## Abstract

Background/Objectives: Elevated gastric residual volume (GRV) is traditionally read as feeding intolerance, and randomized trials in general intensive care units (ICUs) have discredited its routine use. Whether this holds in neurocritical patients is unknown. Methods: This exploratory, hypothesis-generating single-center retrospective cohort study comprised 355 neurosurgical ICU adults with documented GRV (2022–2026)—the exposure was a peak GRV ≥ 250 mL, the institutional feeding-hold threshold (*n* = 32). Three explanations were examined: prognosis (Firth penalized regression for in-hospital mortality), nutrition (standardized mean differences with causal mediation), and intracranial pathology (rank-based partial correlation between patient-level maxima of GRV and intracranial pressure (ICP) in the same early ICU window, plus trajectory phenotyping). Results: Elevated GRV was not significantly associated with in-hospital mortality (adjusted odds ratio 1.46; 95% confidence interval (CI) 0.52–3.65) and was not associated with greater deterioration in laboratory-based systemic inflammatory/nutritional indices than in comparators, with null mediation. The patient-level maxima of GRV and ICP were correlated before adjustment (ρ = 0.28) and 0.19 after full adjustment (95% CI −0.001 to 0.37, *p* = 0.051; 10 exposed of 109), and intracranial hypertension (ICP > 22 mmHg) occurred in 90% of exposed versus 61% of unexposed monitored patients. A high-GRV trajectory phenotype was the youngest class yet showed the most frequent intracranial hypertension, although its membership and persistence were highly sensitive to missing-data handling. Conclusions: In neurocritical patients receiving enteral tube feeding with documented GRV monitoring, elevated GRV was not associated with greater deterioration in laboratory-based systemic inflammatory/nutritional indices, whereas a possible association with higher early ICP was observed in a small, monitored subset. Because the ICP-monitored subset was small, these exploratory, hypothesis-generating findings do not establish a brain–gut signal. They raise the hypothesis that elevated GRV in this setting may reflect intracranial rather than solely gastrointestinal processes and require prospective confirmation with temporally paired measurements.

## 1. Introduction

Early enteral nutrition is a cornerstone of care for critically ill patients [1], and gastric residual volume (GRV) has historically been monitored as an indicator of gastric emptying and feeding tolerance [2]. In clinical practice, an elevated GRV has been interpreted as evidence of delayed gastric emptying warranting reduction or interruption of enteral feeding, and this interpretation has shaped bedside decisions across a wide range of intensive care populations.

The clinical value of routine GRV monitoring, however, has been substantially reappraised over the past decade. In the general intensive care setting, the NUTRIREA-1 trial demonstrated that omitting GRV monitoring did not increase ventilator-associated pneumonia and was accompanied by improved achievement of caloric targets [3]. The REGANE trial similarly reported that raising the interruption threshold from 200 mL to 500 mL did not compromise safety [4]. On the basis of this evidence, contemporary guidelines from ASPEN/SCCM [5], ESPEN [1], and ESICM [6] have moved away from routine GRV-triggered feeding holds in the general intensive care unit (ICU). Thresholds nonetheless remain heterogeneous in practice, ranging from 200 to 500 mL. These recommendations rest on the implicit premise that an elevated GRV reflects a gastrointestinal event—delayed gastric emptying or feeding intolerance—that need not itself alter outcome [2].

Whether the same premise holds in neurocritical care is less certain. Patients with acute intracranial pathology are exposed to several central mechanisms that may delay gastric emptying independent of primary gastrointestinal disease. Sustained intracranial hypertension is itself a determinant of sympathetic activity [7] and acquired brain injury can produce a broader syndrome of paroxysmal sympathetic hyperactivity [8]. Both of these central states can inhibit gastric motility via a central rather than a peripheral mechanism. Neurotrauma has also been shown to alter vagal afferent signaling from the gastrointestinal tract, disrupting the vago-vagal reflex circuit that regulates gastric motility [9]. Consistent with these mechanisms, patients with moderate to severe head injury exhibit prolonged gastric emptying relative to healthy controls [10], and feeding intolerance—of which elevated GRV is the leading manifestation—affects a substantial proportion of neurocritically ill patients [11]. If so, GRV in this population may reflect intracranial as well as gastrointestinal processes, and its meaning would differ from that in the general ICU.

This alternative interpretation has not been rigorously examined. First, existing studies of GRV in neurocritically ill patients have focused on feeding intolerance and caloric-target achievement, treating GRV as a nutritional variable rather than as a candidate index of intracranial state [11]. Second, no study to our knowledge has quantitatively compared GRV and intracranial-pressure measurements obtained within the same early ICU window in the same patients. Third, GRV has been analyzed almost exclusively as a static threshold-based variable, and its longitudinal trajectory over the course of an ICU stay has not been characterized.

We, therefore, sought to examine whether an elevated GRV in neurocritically ill patients behaves less as a marker of gastric intolerance than as a signal of intracranial pathology. We hypothesized that elevated GRV would (i) not be associated with in-hospital mortality, (ii) not be mediated by deterioration in laboratory-based nutritional indices, and (iii) instead be associated with intracranial hypertension within the same early ICU window. Given the observational design and the window-based rather than temporally paired nature of these measurements, we regard these analyses as hypothesis-generating.

## 2. Materials and Methods

### 2.1. Study Design and Setting

This was a single-center, retrospective cohort study conducted at Samsung Medical Center, a tertiary academic hospital in Seoul, South Korea. We reviewed all adult patients (aged ≥ 18 years) admitted to the neurosurgical intensive care unit (NSICU) between January 2022 and April 2026. The study was approved by the Institutional Review Board of Samsung Medical Center (IRB No. SMC 2026-07-047-001), and the requirement for informed consent was waived owing to the retrospective observational design. Clinical data were retrospectively extracted from the hospital’s Clinical Data Warehouse (CDW), “Darwin-C,” an institutional research platform designed for investigators to search and retrieve de-identified medical records from the electronic archives. The CDW contains data pertaining to more than 4 million patients. Clinical, laboratory, and outcome data were extracted from the CDW after finalizing the patient list for this study.

### 2.2. Study Population, Exposure, and Outcomes

In our NSICU, GRV is monitored under a standardized nursing protocol for all enterally tube-fed patients. All patients begin enteral nutrition as continuous feeding, during which GRV is recorded, and GRV continues to be monitored after the transition to intermittent bolus feeding. We defined the analytic cohort as all adults admitted between January 2022 and April 2026 who had at least 1 documented GRV measurement within 18 days of the index NSICU admission (the data source is described below). Patients without any documented GRV measurement did not contribute an observed exposure and were, therefore, not analyzed, avoiding the misclassification that would arise from treating undocumented patients as unexposed. Because this was a hypothesis-generating observational study that used all available data during the documentation-complete era (from January 2022 onward, when structured GRV flowsheet documentation was complete in the data warehouse), no a priori sample-size calculation was performed. The Glasgow Coma Scale (GCS) total score was recalculated from the individual eye, verbal, and motor subscores extracted from the neurological assessment records. For patients in whom the verbal component was unassessable owing to endotracheal intubation or tracheostomy, the verbal score was imputed using the Rutledge regression method [12], which predicts the verbal score from the eye and motor subscores via a validated regression equation. GRV was measured by gentle aspiration through the enteral feeding tube using an enema syringe at 4 h intervals during each nursing shift, in both continuous-feeding and intermittent bolus-feeding phases. When the aspirated volume was <250 mL, the aspirate was returned and enteral feeding was continued. When the aspirated volume was ≥250 mL, the aspirate was discarded, enteral feeding was held, and the attending or on-call neurointensivist was notified for a decision on restart. The 250 mL feeding-hold threshold predates the current study and reflects long-standing institutional nursing practice. The Rutledge method is a validated regression-based approach; however, it provides population-level estimates that may not exactly reproduce an individual patient’s unobserved verbal response, and, because intubation is non-random with respect to illness severity, systematic misclassification in imputed values cannot be excluded. APACHE II scores were those recorded at ICU admission in the institutional ICU scoring system. Their GCS component was derived from the clinically recorded GCS total (in 262 of the 267 patients whose verbal response was recorded as unassessable, the recorded total corresponded to the eye and motor scores plus a verbal score of 1) and not from the imputed value. The imputed admission GCS was used for the descriptive baseline characteristics (Table 1), for the descriptive comparison of the trajectory clas-ses (see Section 3.6), and as a covariate only in the exploratory mediation models (Section 2.4). It entered neither the APACHE II score nor the outcome models, in which admission GCS is reflected only through APACHE II. The imputed and recorded admission GCS totals differed in 30 of 351 patients (by 1–2 points), and a sensitivity analysis using the raw GCS motor subscore is described in Section 2.4. Because GRV is monitored under this protocol only in enterally tube-fed patients, a documented GRV served as the operational indicator that enteral tube feeding was in progress. A free-text screen of nursing feeding records identified tube-feeding documentation in 510 of 6067 NSICU patients admitted during the study era (1193 of 14,572 patients across the full 2016–2026 extraction window), yet it captured only 332 of the 355 cohort patients (94%), indicating that free-text feeding documentation is incomplete and unsuitable as an enrollment filter. The cohort was, therefore, defined by documented GRV measurement, and the feeding record screen is presented as contextual information in the study flow diagram (Supplementary Figure S1).

The exposure was defined a priori as a peak GRV ≥ 250 mL, corresponding to the institutional enteral nutrition hold threshold. GRV values were extracted from the ICU nursing flowsheets by time from the index NSICU admission (through day 18). Because the registry extract contained one NSICU admission per patient, readmissions to the NSICU and transfers to other ICUs were not linked. GRV values documented during such subsequent ICU stays within the window were nevertheless captured, and because GRV is not measured on general wards at our institution, all GRV values reflect ICU care. In 83 of the 355 patients some values were documented after the index NSICU stay, and in 52 of these (whose index NSICU stay lasted a median of 0.9 days), all values were documented after it. In one exposed patient the first GRV ≥ 250 mL was recorded after the index NSICU stay. A sensitivity analysis restricting the exposure to values documented during the index NSICU stay is reported in Section 3.3. Patients were classified as exposed (peak GRV ≥ 250 mL) or unexposed (all documented GRV values < 250 mL).

The primary outcome was in-hospital mortality. Secondary outcomes were ICU mortality (death during the index NSICU stay) and 28-day all-cause mortality (death from any cause within 28 days of ICU admission, inclusive). A poor neurological outcome—defined a priori as a Glasgow Outcome Scale (GOS) score of 1–3 (death, persistent vegetative state, or severe disability), with GOS 4–5 (moderate disability or good recovery) considered favorable and determined at hospital discharge—was examined as an exploratory outcome. The discharge GOS category was assigned by a fixed rule, implemented when the study database was constructed and before the present analyses, that was applied sequentially to vital status and to the neurological assessment records closest to hospital discharge (GCS, level of consciousness on the AVPU scale, orientation, and delirium screening). The rule, as applied, and the resulting distribution are given in Supplementary Table S10. Because the rule reflects consciousness and cognitive status rather than a structured assessment of functional independence, we refer to this variable as a rule-derived GOS category, in which the GOS labels denote the numbering of the categories rather than validated functional classes, and it was analyzed only as the binary contrast of GOS 1–3 versus 4–5. For patients with an artificial airway at discharge (*n* = 136), the GCS used for this assignment incorporated the Rutledge-imputed verbal score, and sensitivity analyses of this outcome under alternative handling of these patients are reported in Section 3.3. Intracranial hypertension, defined as an intracranial pressure (ICP) exceeding 22 mmHg at any point during ICP monitoring, in accordance with the treatment threshold recommended in the Brain Trauma Foundation guidelines [13], served as the mechanistic variable linking GRV to intracranial pathology.

### 2.3. Covariates and Data Collection

Baseline characteristics collected at ICU admission included demographics (age and sex), ICU admission diagnosis, illness severity (corrected GCS, Acute Physiology and Chronic Health Evaluation (APACHE) II score [14], and Sequential Organ Failure Assessment (SOFA) score [15]), nutritional indices (serum albumin, absolute lymphocyte count (ALC), the Prognostic Nutritional Index (PNI) [16], the Controlling Nutritional Status (CONUT) score [17], and the modified Nutrition Risk in Critically Ill (mNUTRIC) score [18]), comorbidities (hypertension, diabetes mellitus, malignancy, chronic kidney disease, and chronic liver disease), and ICU management variables (duration of mechanical ventilation, ICP monitoring, continuous renal replacement therapy (CRRT), osmotherapy, and vasopressor or inotrope use). Osmotherapy was recorded as any osmotherapy and separately as mannitol and hypertonic saline. Nutritional indices were assessed at ICU admission, at ICU discharge (the end of the index NSICU stay), and as the admission-to-discharge change (Δ). The reason for NSICU admission (admission diagnosis) was classified into seven categories and included as a covariate in the adjusted Firth logistic regression models.

### 2.4. Statistical Analysis

Continuous variables are presented as median (interquartile range (IQR)) and were compared using the Mann–Whitney U test. Categorical variables are presented as *n* (%) and were compared using Fisher’s exact test or the chi-square test, as appropriate. Standardized mean differences (SMDs) were used to compare nutritional indices between exposure groups, with |SMD| < 0.2 considered a trivial difference. Because deaths among exposed patients were few, the association between GRV exposure and each outcome was estimated primarily by Firth penalized logistic regression [19], which reduces small-sample bias, with crude odds ratios (ORs) reported in parallel in accordance with the Strengthening the Reporting of Observational Studies in Epidemiology (STROBE) reporting guideline [20]. Models were adjusted for age, APACHE II score, and admission diagnosis. Admission GCS was not entered separately because it is a component of the APACHE II score, and models were kept parsimonious given the limited number of events. GCS and APACHE II were entered together only in the exploratory mediation models described below. Variance inflation factors for the non-diagnostic covariates were ≤1.3 in the outcome models (adjusted generalized variance inflation factor for the admission diagnosis block, 1.01) and in the partial-correlation models, and ≤2.3 in the mediation models. Robustness of the mortality estimate was examined by adding mechanical ventilation and osmotherapy as covariates. Baseline characteristics of patients with versus without invasive ICP monitoring were compared to characterize the monitored subset (Supplementary Table S1), and the distribution of individual GOS scores (1–5) by exposure was tabulated descriptively (Supplementary Table S2). GRV trajectory phenotypes were derived from daily peak GRV across ICU days 0–14, modeled by longitudinal K-means clustering (time-series K-means) [21] of log(1 + GRV) values (four classes specified a priori for clinical interpretability; Euclidean metric; 30 random initializations; random seed 42), restricted to patients with at least three measurement days and a first measurement by ICU day 5, with within-patient missing days imputed by linear interpolation combined with last- and first-observation carry-forward and carry-backward. Class labels were ordered ascending by class-median peak GRV so that Class 1 represented the lowest-GRV phenotype, and between-class comparisons used the Kruskal–Wallis test for continuous variables and the chi-square test for categorical variables. The robustness of the trajectory phenotypes to the pre-specified number of classes was examined by repeating the clustering with K = 3 and K = 5 under otherwise identical settings (Supplementary Table S3 and Figure S2). Because these inclusion criteria excluded patients with the earliest deaths and the shortest ICU stays, the trajectory findings are interpreted as complementary to, rather than a replacement for, the primary categorical analysis—the composition of included versus excluded patients is summarized in Section 3.6. Among the 213 patients included in the trajectory analysis, the median number of observed GRV measurement days per patient was 7 (IQR 5–11, minimum 3), corresponding to a maximum of 12 imputed days per patient within the 15-day observation window (ICU days 0–14). The minimum-measurement criterion was applied within this window, and no upper limit on the proportion of imputed days was imposed. Interpolation and carry-forward/backward were performed on the log scale, and carried values could extend beyond the last observation. Among the 213 patients, 1657 of 3195 patient-days (52%) had an observed value and 1538 (48%) were imputed: 981 (31%) were carried forward after the last observed day, 458 (14%) were carried backward before the first observed day, and 99 (3%) were interpolated between observations. The last observed GRV value defined the end of the observed series—it did not necessarily coincide with ICU discharge or death, because documentation also ends when enteral feeding is stopped or oral intake resumes. The last observation preceded day 14 in 155 patients (73%; median last observed day 9). The index NSICU stay recorded in the registry ended before day 15 in 165 patients (78%), but this boundary was not used because ICU readmissions were not linked. In the previous version of this manuscript, the statement that 25 patients died or left the ICU within the window referred to death or hospital discharge, understated the extent of carry-forward, and is corrected here. To examine the dependence of the phenotypes on this imputation, the trajectory modeling was repeated using observed values only: (i) latent class growth analysis—a finite mixture of cubic polynomial trajectories of log(1 + GRV) with a common residual variance, estimated by expectation–maximization with 60 random starts under a missing-at-random assumption, with K = 4 fixed for comparability with the primary analysis—and (ii) time-series K-means with a dynamic-time-warping distance on series truncated at the last observation, with interpolation confined to gaps between observations (which, because ICU episodes were not linked, could include intervals outside an ICU). Agreement with the primary classes was summarized by cross-tabulation and by the proportion with ICP > 22 mmHg in each class (Section 3.6). A day-7 landmark analysis was performed to evaluate the potential influence of immortal-time bias on the mortality estimate, in which patients who died before ICU day 7 were excluded, the exposure was defined as a first GRV ≥ 250 mL within ICU days 0–7, and subsequent in-hospital mortality was the outcome (Supplementary Table S4). The mortality and neurological outcome models were also refitted with the exposure restricted to GRV values documented during the index NSICU stay, excluding patients without such values, and the poor-neurological-outcome model was refitted under alternative handling of patients with an artificial airway at discharge (classifying such survivors as favorable only if the eye and motor responses were maximal and, as an extreme assumption, classifying all such survivors as poor outcome). In addition, sensitivity analyses were performed in which the raw GCS motor subscore (available in 351 of 355 patients) replaced the APACHE II score as the severity adjustment in the outcome models (Supplementary Table S5). The association between peak GRV and peak ICP over the early ICU window was assessed by the Spearman rank correlation and by rank-based partial correlation adjusted sequentially for opioid exposure and then for age, diabetes mellitus, and vasopressor use. Because rank-based partial correlation is robust to the skewed distribution of GRV, it was preferred over ordinary least squares. Partial-correlation *p*-values were computed from t distributions with *n* − k − 2 degrees of freedom, where k is the number of conditioning covariates, and 95% confidence intervals used the Fisher z transformation. Opioid exposure was modeled primarily as a binary indicator of any parenteral opioid during the peri-admission window (ICU days −3 to +7) and, in sensitivity analyses, as intravenous morphine milligram equivalents (MME) and log-transformed MME (Supplementary Table S6). Because osmotherapy is a near-universal treatment of intracranial hypertension in this setting and is administered in response to elevated ICP, adjusting for it may introduce collider or overadjustment bias. It was, therefore, examined only as a sensitivity adjustment (Supplementary Table S7). The dichotomous relationship between GRV ≥ 250 mL and ICP > 22 mmHg was additionally estimated by Firth regression with the same covariates. Because the ICU discharge laboratory values refer to the index NSICU stay, the comparison of nutritional indices and the mediation analyses were repeated in the 303 patients whose GRV was documented during that stay. To assess whether the association between GRV ≥ 250 mL and poor neurological outcome was mediated by a change in laboratory-based nutritional indices, causal mediation analysis (Imai–Tingley quasi-Bayesian approximation [22], 2000 Monte Carlo simulations; outcome and mediator models adjusted for age, sex, APACHE II, SOFA, and the corrected admission GCS) estimated the average causal mediation effect (ACME) and the average direct effect (ADE) for ΔPNI and ΔCONUT as candidate mediators. These mediation analyses are exploratory: the exposure summarizes the entire ICU stay, while the mediators are admission-to-discharge changes, so strict temporal ordering of exposure and mediator cannot be guaranteed. All statistical tests were two-sided, and *p* < 0.05 was considered statistically significant. Analyses were performed using Python 3.11 (Python Software Foundation, Wilmington, DE, USA) with the pandas (version 3.0.2), NumPy (2.4.4), SciPy (1.17.1), statsmodels (0.15.0), scikit-learn (1.8.0), and tslearn (0.9.0) libraries.

## 3. Results

### 3.1. Study Population

During the study period, 355 adults admitted to the NSICU between 2022 and 2026 had at least one documented GRV measurement and constituted the analytic cohort (Supplementary Figure S1). Thirty-two patients (9.0%) had a peak GRV ≥ 250 mL and 323 (91.0%) did not. Within the cohort, the median age was 64 years (IQR 52–74) and the median admission corrected GCS was 3 (IQR 3–6), reflecting a deeply sedated or unconscious population—276 patients (77.7%) received mechanical ventilation and 146 (41.1%) underwent ICP monitoring. Fifty-four patients (15.2%) died in hospital. The GRV–ICP mechanism sub-cohort comprised 109 patients with both documented GRV and invasive ICP monitoring over ICU days 0–7, and the trajectory sub-cohort comprised 213 patients.

### 3.2. Baseline Characteristics

Baseline characteristics by GRV exposure are presented in Table 1. Rather than being frailer, exposed patients were younger (median 54.5 vs. 65.0 years, *p* = 0.024) and more often male (75.0% vs. 52.6%, *p* = 0.016). Illness severity was comparable between groups: the APACHE II score (28.5 vs. 26.0, *p* = 0.095), SOFA score (*p* = 0.247), and admission GCS (*p* = 0.194) did not differ significantly. Baseline laboratory-based nutritional indices were likewise comparable, with no significant difference in serum albumin (3.65 vs. 3.50 g/dL, *p* = 0.373), ALC, PNI, CONUT score, or the proportion at high nutritional risk (mNUTRIC ≥ 5, 43.8% vs. 43.3%, *p* = 1.000); where indices differed, the direction favored the exposed group. Brain tumor was the most common reason for admission in both groups, whereas traumatic brain injury was more frequent among exposed patients (25.0% vs. 8.4%). Consistent with a greater treatment burden, the exposed group more often received mechanical ventilation (96.9% vs. 75.9%, *p* = 0.003) and tended toward a longer duration of ventilation (13.0 vs. 10.0 days, *p* = 0.056), while ICP monitoring was numerically more frequent (50.0% vs. 40.2%, *p* = 0.347).

### 3.3. Clinical Outcomes

In-hospital mortality did not differ significantly by GRV exposure. In-hospital death occurred in 6 of 32 exposed patients (18.8%) versus 48 of 323 comparators (14.9%), corresponding to a crude OR of 1.39 (95% confidence interval (CI) 0.56–3.46) and a Firth-adjusted OR of 1.46 (95% CI 0.52–3.65, *p* = 0.449) after adjustment for age, APACHE II, and admission diagnosis (Table 2). The adjusted OR was 1.26 after adding mechanical ventilation and 1.45 after adding osmotherapy (Supplementary Table S8). ICU mortality (4 of 32 (12.5%) vs. 20 of 323 (6.2%); adjusted OR 2.26, 95% CI 0.62–7.03, *p* = 0.203) and 28-day mortality (6 of 32 (18.8%) vs. 38 of 323 (11.8%); adjusted OR 1.97, 95% CI 0.69–5.04, *p* = 0.194) also did not differ significantly. In a day-7 landmark analysis excluding the 11 patients who died before ICU day 7 (344 at risk; exposure, first GRV ≥ 250 mL within days 0–7, *n* = 20), in-hospital death occurred in 4 of 20 exposed versus 39 of 324 unexposed patients (Firth-adjusted OR 2.02, 95% CI 0.58–5.99, *p* = 0.250; Supplementary Table S4). Restricting the exposure to GRV values documented during the index NSICU stay and excluding the 52 patients without such values (300 patients with complete covariates; 31 exposed) gave a Firth-adjusted OR of 1.51 (95% CI 0.50–4.02, *p* = 0.443) for in-hospital mortality and 5.36 (95% CI 2.27–13.79) for poor neurological outcome. A poor neurological outcome (GOS 1–3) occurred in 23 of 32 exposed (71.9%) versus 136 of 323 unexposed patients (42.1%; crude OR 3.40, 95% CI 1.55–7.45; Firth-adjusted OR 4.84, 95% CI 2.11–12.08). In the breakdown of individual GOS scores at hospital discharge (Supplementary Table S2), this association was driven primarily by the severe-disability category (GOS 3, i.e., survivors with a discharge GCS of 6–12 or with impaired orientation: 50.0% vs. 24.5%) rather than by death (GOS 1: 18.8% vs. 14.9%) or persistent vegetative state (GOS 2: 3.1% vs. 2.8%). The association with poor neurological outcome was similar when survivors with an artificial airway at discharge were classified as favorable only if their eye and motor responses were maximal (adjusted OR 4.52, 95% CI 1.97–11.31) and when all such survivors were classified as poor outcome (adjusted OR 4.12, 95% CI 1.67–11.62). A baseline comparison between patients who underwent invasive ICP monitoring (*n* = 146) and those who did not (*n* = 209) is presented in Supplementary Table S1.

### 3.4. GRV and Nutritional Status

**Figure 1:**
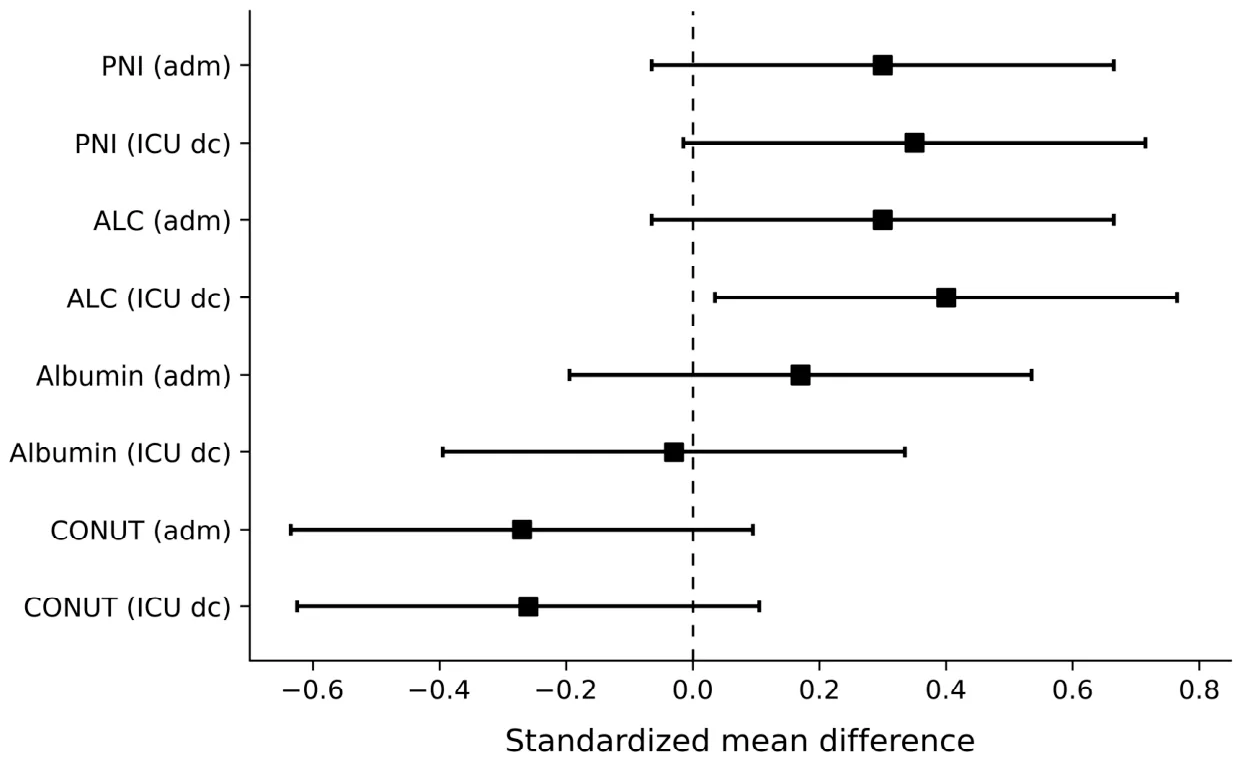
Laboratory-based nutritional indices by GRV exposure. Forest plot of standardized mean differences (SMDs; GRV ≥ 250 minus < 250) for four nutritional indicators at admission and at ICU discharge. Positive SMD = higher raw value in GRV ≥ 250. Error bars represent 95% confidence intervals. Values around zero and confidence intervals crossing the reference line indicate no meaningful between-group difference in these indices. SMD, standardized mean difference; ALC, absolute lymphocyte count; PNI, Prognostic Nutritional Index; CONUT, Controlling Nutritional Status.

At ICU admission, laboratory-based nutritional indices did not differ significantly between exposure groups (all *p* > 0.1; Figure 1). At ICU discharge, the GRV ≥ 250 group had a higher ALC (SMD +0.40) and PNI (SMD +0.35) and a similar serum albumin (SMD −0.03). Between-group differences in admission-to-discharge change were small for every index (all |SMD| ≤ 0.13). In causal mediation analysis of poor neurological outcome, the average causal mediation effect (ACME) through ΔPNI was +0.001 (*p* = 0.95) and through ΔCONUT +0.007 (*p* = 0.74), while the average direct effect (ADE) remained significant (*p* = 0.016 and 0.008, respectively). In the 303 patients whose GRV was documented during the index NSICU stay (31 exposed), the results were similar: at ICU discharge the SMDs were −0.01 for albumin, +0.33 for ALC, +0.31 for PNI, and −0.19 for the CONUT score, the between-group differences in admission-to-discharge change remained small (all |SMD| ≤ 0.13), and the ACME was +0.003 (*p* = 0.89) through ΔPNI and +0.013 (*p* = 0.56) through ΔCONUT, with the ADE remaining significant (*p* = 0.008 and 0.003).

### 3.5. GRV and Intracranial Pressure

**Figure 2:**
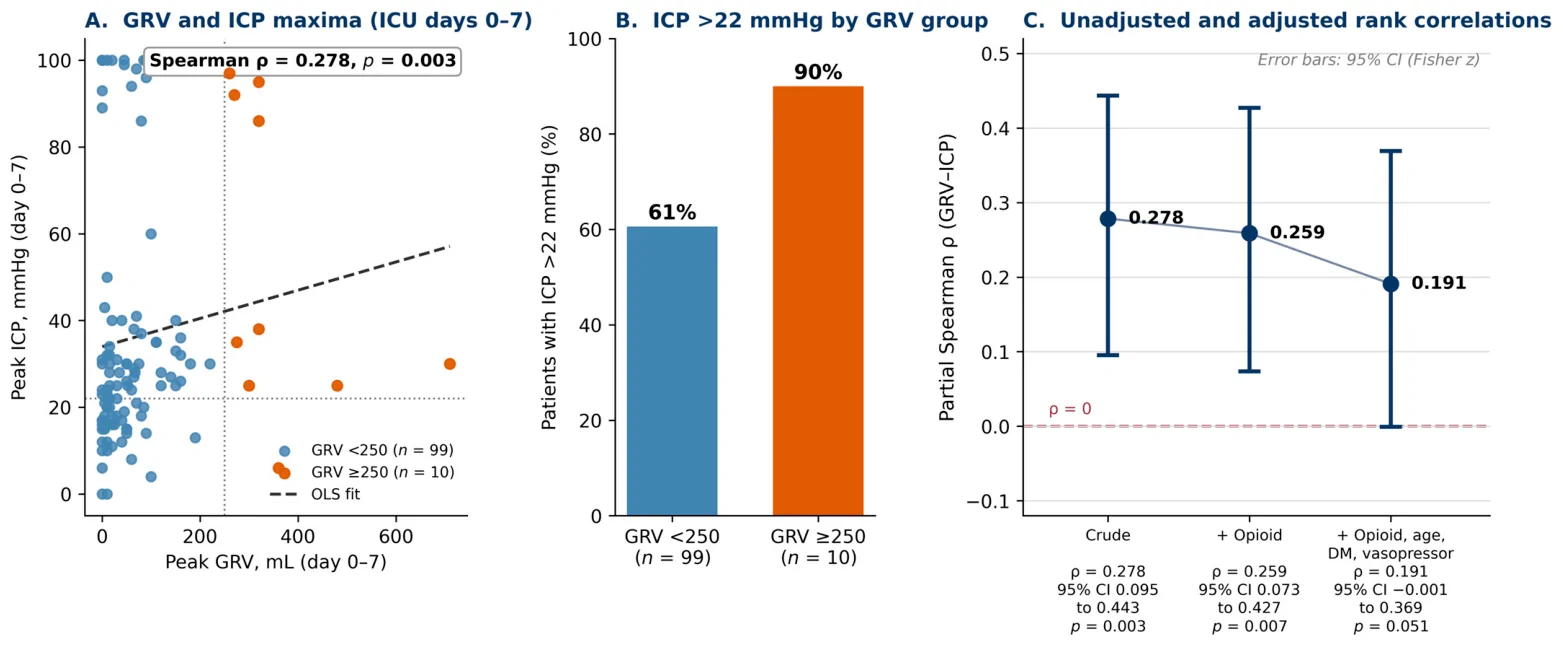
Association between patient-level maxima of GRV and ICP within the same early ICU window (days 0–7) as a candidate brain–gut signal. (**A**) Patient-level maximum GRV vs. patient-level maximum ICP within ICU days 0–7 (*n* = 109; 10 exposed, 99 unexposed) with OLS trend line (Spearman ρ = 0.278, *p* = 0.003). Dotted lines in (A) mark the exposure threshold (peak GRV 250 mL, vertical) and the intra-cranial hypertension threshold (ICP 22 mmHg, horizontal). (**B**) Intracranial hypertension (ICP > 22 mmHg) by GRV group (60/99 (61%) vs. 9/10 (90%)). (**C**) Unadjusted and adjusted partial Spearman correlations (error bars, 95% confidence intervals; point estimates, confidence limits, and *p*-values are printed beneath each specification). The fully adjusted estimate was 0.191 (95% CI −0.001 to 0.37, *p* = 0.051). GRV, gastric residual volume; ICP, intracranial pressure; OLS, ordinary least squares.

Among the 109 patients with both documented GRV and invasive ICP monitoring over ICU days 0–7 (10 exposed, 99 unexposed), the patient-level maximum GRV and the patient-level maximum ICP within this same early ICU window were correlated (Spearman ρ = 0.278, *p* = 0.003; Figure 2A). These two maxima did not necessarily occur on the same calendar day, so this analysis reflects an association between patient-level intensity summaries within the same early ICU window rather than a day-to-day temporal coupling. Accordingly, this analysis captures patient-level severity summaries within a concurrent timeframe rather than direct temporal lead–lag coupling. Intracranial hypertension (ICP > 22 mmHg) occurred in 9 of 10 exposed (90%) versus 60 of 99 unexposed (61%) patients (Figure 2B). The partial correlation was 0.259 after adjustment for opioid exposure (*p* = 0.007) and 0.191 after further adjustment for age, diabetes, and vasopressor use (95% CI −0.001 to 0.37, *p* = 0.051; Figure 2C), which did not reach statistical significance—it ranged from 0.259 to 0.242 across binary, MME, and log-MME opioid specifications (all *p* ≤ 0.012; Supplementary Table S6). After adjustment for osmotherapy (near universal in this window, 98%), the partial correlation was 0.198 (*p* = 0.044; Supplementary Table S7). In the dichotomous analysis, the corresponding adjusted estimate was imprecise and did not reach statistical significance (Firth-adjusted OR 4.77, 95% CI 0.74–110.5, *p* = 0.065). The wide confidence interval reflects the small exposed sub-sample and precludes a definitive inference.

### 3.6. GRV Trajectory Phenotypes and the Class 4 Paradox

**Figure 3:**
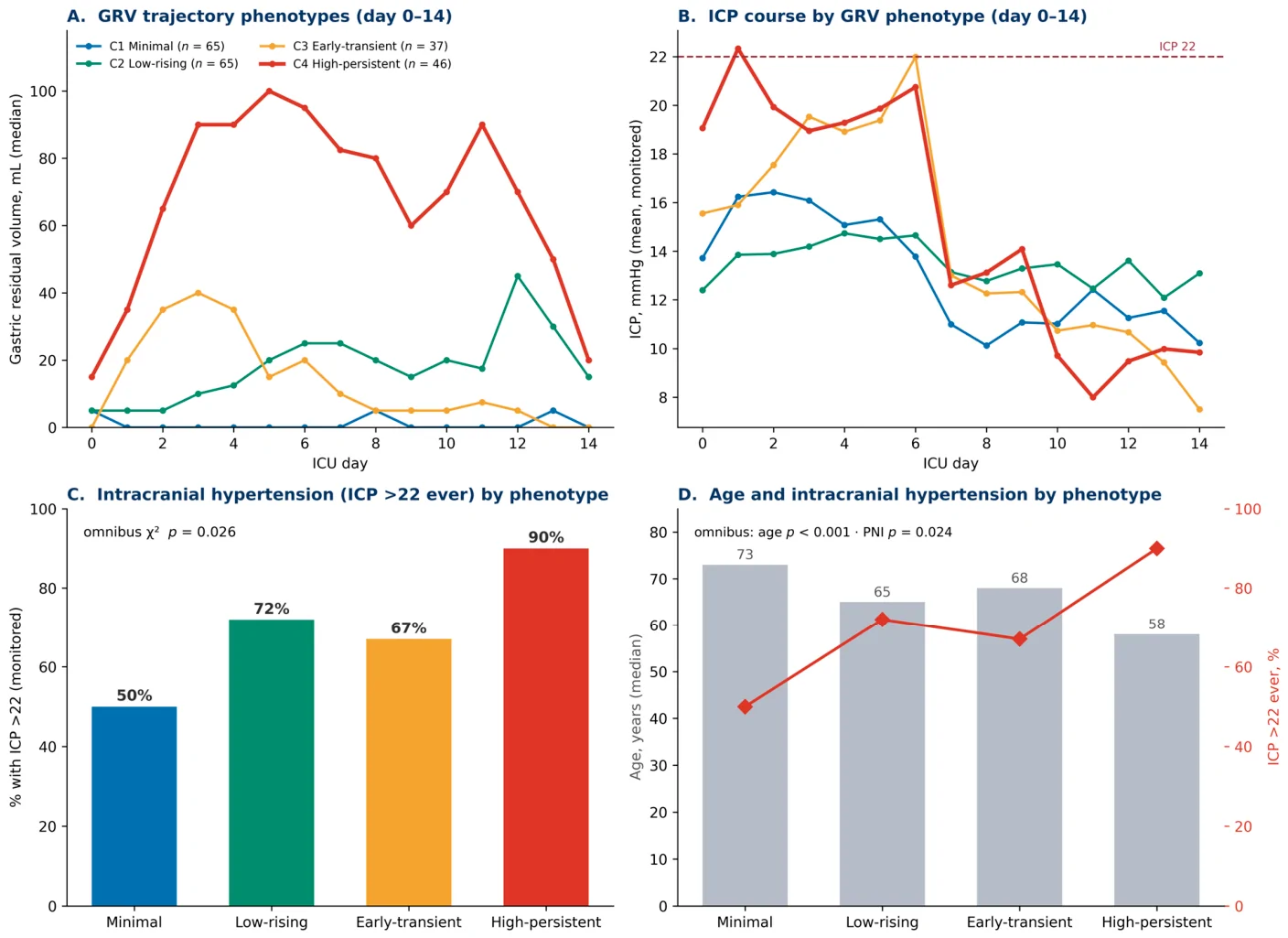
GRV trajectory phenotypes and their intracranial-pressure course. (**A**) Longitudinal K-means clustering of daily GRV identifies four trajectory phenotypes (*n* = 213). (**B**) Mean ICP course by phenotype. (**C**) The proportion of monitored patients with ICP > 22 mmHg differed across phenotypes and was highest in C4 (omnibus χ^2^ *p* = 0.026). (**D**) The youngest phenotype (C4) shows the most frequent intracranial hypertension despite the most favorable admission laboratory-based nutritional indices. ICP, intracranial pressure.

Longitudinal K-means clustering of daily GRV over ICU days 0–14 identified 4 phenotypes among 213 patients (Table 3, Figure 3): Class 1 (minimal, *n* = 65), Class 2 (low-rising, *n* = 65), Class 3 (early-transient, *n* = 37), and Class 4 (high-persistent, *n* = 46), differing in median peak GRV (20, 70, 80, and 200 mL, respectively; overall *p* < 0.001). Compared with the 142 patients excluded by the trajectory criteria, the 213 included patients had similar in-hospital mortality (14.1% versus 16.9%, *p* = 0.55) and age (median 65 versus 63 years, *p* = 0.25) but higher APACHE II scores (median 27 versus 25, *p* < 0.001), reflecting exclusion of both the earliest deaths and short-stay, less severely ill admissions. The four classes differed significantly in age, sex, admission nutritional indices (albumin, PNI, and CONUT), peak GRV, and the proportion with intracranial hypertension (omnibus Kruskal–Wallis or chi-square tests, all *p* < 0.05; Table 3), whereas illness-severity scores and outcomes did not. Class 4 (*n* = 46) had the lowest median age (58 years, versus 73 years in Class 1), the highest admission PNI (43.9), the lowest CONUT score (2.0), the numerically lowest proportion at high nutritional risk (mNUTRIC ≥ 5, 37%; omnibus *p* = 0.27), and the highest proportion with intracranial hypertension among monitored patients (ICP > 22 mmHg in 18 of 20 monitored Class 4 patients (90%), versus 50% in Class 1; omnibus *p* = 0.026). Mortality and poor neurological outcome did not differ significantly across phenotypes. Sensitivity clustering with K = 3 and K = 5 (Supplementary Figure S2 and Table S3) consistently identified the high-persistent GRV phenotype irrespective of the specified class number, supporting the robustness of this phenotype to the choice of K.

In sensitivity analyses that used only observed GRV values, without carry-forward beyond the last observation (Supplementary Table S9 and Figure S3), both approaches identified a high-GRV class. The latent-class-growth high-GRV class comprised 20 patients, all of whom belonged to primary Class 4 (20 of 46 retained), and the DTW-based high-GRV class comprised 36 patients, 27 of whom belonged to primary Class 4 (9 were drawn from Classes 2 and 3). Class size and membership were, therefore, sensitive to the handling of missing data and to the modeling approach. Intracranial hypertension was observed in 12 of 12 and 15 of 16 monitored patients in these classes, the highest proportion of any class in each analysis, but these are small and overlapping monitored subsets. Classification uncertainty in the latent class model was moderate (entropy 0.69; maximum posterior probability < 0.7 in 64 of 213 patients).

## 4. Discussion

In this single-center retrospective cohort of 355 adults admitted to an NSICU with documented GRV monitoring, a peak GRV of at least 250 mL was not significantly associated with in-hospital mortality, although it was associated with poorer neurological status at hospital discharge. Between-group differences in the change of the measured laboratory-based systemic inflammatory/nutritional indices were small, and exploratory causal mediation through nutritional change was essentially null. Peak GRV instead showed a possible association with peak intracranial pressure across the first ICU week (fully adjusted ρ = 0.19, 95% CI −0.001 to 0.37, *p* = 0.051) that persisted after opioid adjustment, and intracranial hypertension was more frequent in the exposed group. Because only 10 exposed patients had ICP monitoring, these mechanistic findings are exploratory and preliminary. A high-persistent GRV trajectory phenotype was the youngest of the four longitudinal classes, had the most favorable admission laboratory-based nutritional indices, and yet showed the most frequent intracranial hypertension, although its membership and the persistence of its elevation were highly sensitive to the handling of missing trajectory data (Section 3.6).

The absence of an association between peak GRV and in-hospital mortality has to be interpreted in the context of a modest number of exposed events. Formal post hoc power calculation is uninformative once a study is completed, and we, therefore, report the estimate with its confidence interval. This null result is concordant with randomized evidence from general ICU populations in which the routine use of GRV as a trigger for changes in enteral feeding has not translated into mortality differences [3,4]. Selectivity is nonetheless of interest: the same exposure that showed no mortality signal was associated with a proximal neurological outcome and with the intracranial-pressure endpoint, a pattern that would be consistent with, but does not establish, an exposure reflecting intracranial rather than global illness burden. In keeping with this interpretation, the individual GOS breakdown (Supplementary Table S2) showed that the association was driven by survivors in the severe-disability category (impaired consciousness or orientation at discharge) rather than by death.

Elevated GRV was not associated with greater deterioration in the measured laboratory-based systemic inflammatory/nutritional indices than in comparators. Nutritional indices at ICU admission did not differ by exposure, between-group differences in admission-to-discharge change were small for every index, and exploratory causal mediation through either change in PNI [16] or change in CONUT score [17] was essentially null while the direct effect remained [22,23]. At ICU discharge, the exposed group’s ALC and PNI were in fact directionally higher than those of comparators. Because the exposed group was younger by roughly ten years and the high-persistent trajectory class by roughly fifteen years, this direction is best interpreted as residual confounding by age-related nutritional reserve rather than as a distinct effect of the exposure, and the internal consistency of the age signal supports rather than undermines the primary inference.

We recognize that the nutritional indices we employed—serum albumin, absolute lymphocyte count, PNI, and CONUT—are influenced by systemic inflammation, capillary leak, and fluid resuscitation during acute critical illness, and are, therefore, not pure measures of nutritional status [24]. Direct measures of enteral nutrition delivery, such as the percentage of caloric target achieved, daily protein intake, and feeding-interruption days, were not available in our data warehouse extraction. Our findings should, therefore, be interpreted as showing no greater deterioration in the selected laboratory-based systemic inflammatory/nutritional indices than in comparators, which is distinct from overall nutritional status, caloric adequacy, or the broader clinical concept of feeding intolerance.

Peak GRV instead showed a possible association with peak intracranial pressure within the same early ICU window. The association was modestly attenuated by adjustment: it persisted after opioid adjustment (ρ = 0.26, *p* = 0.007) and was 0.19 after full covariate adjustment (95% CI −0.001 to 0.37, *p* = 0.051), and intracranial hypertension was more frequent among patients with an elevated GRV than among comparators. Several central mechanisms can plausibly link these two signals: suppression of vagal outflow by sustained intracranial hypertension [7], disruption of the vago-vagal reflex circuit by neurotrauma-induced changes in vagal afferent signaling [9], and sympathetic activation as part of the Cushing response and paroxysmal sympathetic hyperactivity [7,8]. Each of these mechanisms is expected to delay gastric emptying independent of primary gastrointestinal disease, and each is consistent with the clinical observation that patients with moderate-to-severe head injury exhibit prolonged gastric emptying [10]. Because our measurements were obtained within the same early ICU window rather than as day-by-day temporally paired observations, the analysis captures patient-level severity summaries within a concurrent timeframe rather than direct temporal lead–lag coupling. We, therefore, make no predictive claim about temporal order, and describe GRV as being associated with, not preceding, intracranial pressure. We did not attempt a day-by-day paired analysis for three active reasons: (a) the calendar day on which invasive ICP monitoring was initiated varied considerably across patients, so the sets of days available for pairing would differ from patient to patient, (b) GRV is measured intermittently every 4 h per nursing protocol, whereas ICP is continuously monitored, so per-day summaries are not directly comparable, and (c) ICP is treated as soon as it rises, which could bias any day-level ICP–GRV pairing toward the null and mislead directional inference. Any apparent day-to-day lead that would arise from the delayed placement of intracranial-pressure monitors after admission is thus treated as a measurement artefact rather than as evidence of direction.

Longitudinal K-means clustering of daily GRV over the first two ICU weeks [21] disclosed a phenotype—the high-persistent class—that could not have been recovered from a single peak-based classification. Members of this class were the youngest and had the most favorable admission laboratory-based nutritional indices and yet showed the highest observed proportion of intracranial hypertension, with ICP > 22 mmHg in 18 of 20 monitored patients. The trajectory cohort required at least three days of measurement and a first measurement by ICU day 5, which excluded the earliest deaths (in-hospital mortality 14.1% among included versus 16.9% among excluded patients). The direction of any resulting selection effect cannot be established with certainty, which justifies interpreting the trajectory findings as complementary to, rather than a replacement for, the primary categorical analysis. Sensitivity clustering at K = 3 and K = 5 (Supplementary Figure S2 and Table S3) consistently identified the high-persistent phenotype irrespective of the specified class number, supporting its robustness to the choice of K. When the trajectory modeling was restricted to observed GRV values, a high-GRV class was again identified and again showed the highest proportion of intracranial hypertension, but its size and membership differed from the primary Class 4 (Section 3.6). A relatively high-GRV group was thus identified in each analysis, whereas class membership and the persistence of the elevation were highly sensitive to the imputation method (of the 46 primary Class 4 members, 20 were retained by the latent class model and 27 by the DTW analysis) and were not established as robust.

Several alternative explanations for the observed association merit consideration. Sedative and opioid exposure can slow gastric motility [25], but adjustment for opioids—whether specified as a binary indicator, as continuous MME, or on a logarithmic scale—attenuated the partial correlation only marginally (ρ = 0.259, 0.250, and 0.242, respectively; all *p* ≤ 0.012). Age and cerebral atrophy might plausibly modify the association, but the high-persistent phenotype was the youngest of the four classes, which argues against age as the sole explanation for the observed direction, although residual confounding cannot be excluded. Analyses that required intracranial-pressure monitoring were restricted to a monitored subset and are, therefore, subject to a degree of selection, and the trajectory cohort’s minimum-measurement requirement excludes the earliest deaths, both of which we acknowledge. Because the exposure could not be assigned before some ICU time had elapsed, a component of immortal-time bias is possible [26]. Its expected direction is to attenuate any mortality association, which is consistent with the observed null. To address this concern directly, we performed a day-7 landmark analysis restricted to patients alive at ICU day 7, with exposure defined as reaching a first GRV ≥ 250 mL within the landmark window (Section 3.3, Supplementary Table S4). The landmark estimate was imprecise and non-significant, and consistent in direction with the primary analysis. While this analysis addresses exposure classification within a fixed early window, it cannot establish the absence of a mortality association or fully exclude time-related bias.

Osmotherapy and fluid resuscitation deserve separate consideration as potential treatment-related drivers of impaired gastric motility that are independent of central intracranial signaling. Osmotherapy was administered to 91.8% of the cohort and to 98% of the ICP-monitored sub-cohort, so its effects cannot be separated from those of intracranial hypertension in these data. Hypertonic saline and mannitol alter systemic fluid balance and plasma osmolality: hypertonic saline raises serum sodium and osmolality and may produce sustained hypernatremia when given as a continuous infusion, mannitol induces an osmotic diuresis with intravascular volume shifts that may compromise splanchnic perfusion, and the fluid resuscitation that may accompany these therapies can promote bowel-wall edema. In a randomized trial after elective colonic resection, a positive salt and water balance sufficient to cause a 3 kg weight gain delayed gastric emptying and the recovery of gastrointestinal function [27]. Although this evidence comes from a different clinical setting, it supports fluid and sodium balance as a plausible alternative explanation without establishing a specific mechanism in our cohort. Consensus recommendations on gastrointestinal function in intensive care patients likewise list electrolyte disturbances among the correctable conditions that impair motility and advise avoiding fluid over-resuscitation, which predisposes to intra-abdominal hypertension [28]. Adjustment for osmotherapy as any use, hypertonic saline, or mannitol left the partial correlation essentially unchanged (ρ = 0.198, 0.197, and 0.190; Supplementary Table S7), but with near-universal exposure such adjustment is weakly informative and, because osmotherapy is given in response to elevated ICP, may introduce collider bias. We, therefore, regard hyperosmolar therapy and aggressive fluid resuscitation as alternative or contributory explanations that the present data cannot exclude, and a prospective study should record cumulative osmotic and fluid loads together with temporally paired ICP and GRV measurements.

This study has both strengths and limitations. Restricting the analytic cohort to patients with documented GRV avoided the misclassification that would arise from treating undocumented patients as unexposed, the nutritional pathway was examined by exploratory causal mediation rather than by descriptive comparison alone [22,23], and the association analysis was adjusted sequentially for opioid exposure and other covariates, with osmotherapy—a treatment administered in response to elevated ICP, for which adjustment may introduce collider or overadjustment bias—examined only in sensitivity analyses [29]. The study is single-center, retrospective, and drawn from a specialized NSICU, which constrains generalizability. The intracranial-pressure monitoring subset (146 of 355 patients, 41%) was identified by clinicians on the basis of clinical concern for intracranial hypertension. As shown in Supplementary Table S1, monitored patients were younger, had lower admission GCS scores, more often presented with subarachnoid or intracerebral hemorrhage, more often received mechanical ventilation and osmotherapy, and had more favorable baseline nutritional indices than non-monitored patients, whereas APACHE II scores and in-hospital mortality were similar; consequently, the GRV–ICP findings should not be generalized beyond patients whose presentation prompted invasive monitoring. In addition, the mechanistic sub-cohort analysis was based on 109 patients with ICP monitoring in the same early ICU window, only 10 of whom were exposed to GRV ≥ 250 mL within days 0–7, so the mechanistic findings should be interpreted as exploratory and preliminary hypothesis-generating signals rather than as evidence of a brain–gut link, and they are accompanied by wide confidence intervals for the corresponding adjusted estimates. The exposed group is modest in size, which produces wide confidence intervals for categorical estimates. The verbal component of the GCS was imputed by the Rutledge regression method [12] for patients with an artificial airway at admission and at discharge. Although the primary prognostic models used APACHE II rather than GCS directly, a GCS motor sensitivity analysis (Supplementary Table S5) yielded substantially unchanged estimates, and the association with poor neurological outcome persisted under alternative handling of patients with an airway at discharge (Section 3.3), residual imputation-related bias cannot be fully excluded. Because the registry extract contained one NSICU admission per patient, ICU readmissions and transfers to other ICUs were not linked. ICU mortality and the ICU discharge laboratory values, therefore, refer to the index NSICU stay, whereas GRV documentation, which is confined to ICUs at our institution, captured all ICU stays within the observation window. For the 52 patients whose GRV was documented only after the index NSICU stay, the ICU discharge laboratory values thus preceded the GRV exposure. The nutritional comparison and the mediation analyses were, therefore, repeated without these patients, with similar results (Section 3.4). Neurological outcome was assessed at hospital discharge rather than at a standardized interval, such as three to six months after the acute event. Because the timing of discharge varied across patients and is influenced both by length of stay and by the timing of transfer to post-acute care, the discharge assessment may not reflect the eventual level of recovery, and the association with poorer neurological status should be interpreted with this in mind. Standardized functional outcome assessment at three or six months after the acute event (the GOS or extended GOS, or the modified Rankin Scale for cerebrovascular diagnoses) is widely regarded as the reference standard in neurocritical care and would better characterize longer-term recovery than the discharge category used here, which was derived by a rule from neurological assessment records rather than by structured interview and reflects consciousness and cognitive status rather than functional independence. The number of trajectory classes (K = 4) was pre-specified on clinical-interpretability grounds rather than by data-driven optimization. Sensitivity analyses at K = 3 and K = 5 (Supplementary Figure S2 and Table S3) supported the robustness of the high-persistent phenotype, but the choice of K remains a subjective methodological decision. In the trajectory analysis, 48% of patient-days were imputed, including 31% carried forward after the last observed value (which preceded day 14 in 155 of 213 patients) and 14% carried backward before the first observed value, so the fitted trajectories partly reflect imputation. In sensitivity analyses restricted to observed values, a high-GRV class was again identified and showed the highest proportion of intracranial hypertension, but its size and membership differed from the primary Class 4. Class 4 membership and the persistence of its elevation were thus highly sensitive to the imputation method and to the modeling approach (20 of the 46 primary Class 4 members were retained by the latent class model and 27 by the DTW analysis), and the missing-at-random assumption of the latent class model cannot be verified. Missingness is likely to be informative, because observation ceases for reasons related to clinical status: enteral feeding is stopped because of intolerance or because of recovery to oral intake, patients are transferred to a ward or discharged, or patients die. These events can selectively alter the observed sample, but the unobserved GRV values cannot be inferred from the reason that observation ceased; for example, feeding stopped for intolerance need not have been followed by persistently high values, and transfer or discharge does not identify only improving patients. Carrying the last observed value forward after these events may artificially prolong preceding high or low values, and values extrapolated beyond death do not represent a physiological trajectory, so the primary analysis may have overstated the apparent persistence of Class 4 when the last observed value was high (for example, in patients who died or were transferred early). Analyses limited to observed values avoid this extrapolation but remain subject to bias if the probability of continued observation depends on the unobserved GRV values themselves. The net direction of bias in the association between Class 4 and intracranial hypertension cannot be determined from these data (Supplementary Table S9). The mediation analyses are likewise exploratory, as the mediators were admission-to-discharge changes without guaranteed temporal precedence. Finally, the exposure threshold was not optimized on the current data.

## 5. Conclusions

In neurocritically ill patients receiving enteral tube feeding with documented GRV monitoring, elevated GRV was not associated with greater deterioration in the measured laboratory-based systemic inflammatory/nutritional indices than in comparators. Because feeding intolerance and enteral nutrition delivery were not measured directly, this conclusion is limited to those indices and is distinct from the broader clinical concept of feeding intolerance. The relationship between elevated GRV and intracranial pathology within the same early ICU window is hypothesis-generating, was observed only in the small subset with invasive ICP monitoring, and it does not establish a brain–gut signal. These exploratory findings raise the hypothesis that, in this setting, elevated GRV may reflect intracranial rather than solely gastrointestinal processes. Whether an elevated or persistently elevated GRV could inform the appraisal of the intracranial state alongside other clinical and radiographic information is a question for prospective study. We do not translate these observations into a specific numerical trigger for action. The design is observational, the measurements were obtained within the same early ICU window, and the estimates are hypothesis-generating. Prospective, multicenter validation is required before any change in clinical practice. Such studies should incorporate (i) contemporaneous and, where possible, temporally paired measurements of intracranial pressure and gastric residual volume, (ii) direct measures of enteral nutrition delivery, including daily prescribed and delivered caloric and protein intake and the number of feeding-interruption days, (iii) systematic quantification of cumulative opioid exposure (e.g., morphine milligram equivalents), and (iv) standardized long-term neurological outcomes assessed with the Extended Glasgow Outcome Scale [30] and cause-specific mortality as more disease-proximal endpoints.

## Figures and Tables

**Table 1:** Baseline characteristics by GRV exposure.

Variable	Total (*n* = 355)	GRV ≥ 250 (*n* = 32)	GRV < 250 (*n* = 323)	*p*
**Demographics and severity**				
Age, years	64.0 [52.0–74.0]	54.5 [45.8–72.0]	65.0 [53.0–74.0]	0.024
Male sex	194 (54.6)	24 (75.0)	170 (52.6)	0.016
Admission GCS	3.0 [3.0–6.0]	3.0 [3.0–4.5]	3.0 [3.0–7.0]	0.194
APACHE II	27.0 [23.0–30.0]	28.5 [24.8–32.0]	26.0 [23.0–30.0]	0.095
SOFA	4.0 [2.0–6.0]	4.0 [3.0–6.0]	4.0 [2.0–6.0]	0.247
**Reason for NSICU admission, *n* (%)**				
SAH	22 (6.2)	4 (12.5)	18 (5.6)	0.033 ^a^
ICH	59 (16.6)	4 (12.5)	55 (17.0)	
AIS	15 (4.2)	0 (0.0)	15 (4.6)	
TBI	35 (9.9)	8 (25.0)	27 (8.4)	
Brain tumor	128 (36.1)	9 (28.1)	119 (36.8)	
Cerebrovascular, other	17 (4.8)	2 (6.2)	15 (4.6)	
Other	79 (22.3)	5 (15.6)	74 (22.9)	
**Comorbidities, *n* (%)**				
Hypertension	119 (33.5)	9 (28.1)	110 (34.1)	0.561
Diabetes mellitus	97 (27.3)	4 (12.5)	93 (28.8)	0.060
Malignancy	206 (58.0)	16 (50.0)	190 (58.8)	0.353
Chronic kidney disease	29 (8.2)	2 (6.2)	27 (8.4)	1.000
Chronic liver disease	7 (2.0)	1 (3.1)	6 (1.9)	0.487
**Laboratory-based nutritional indices at admission**				
Albumin (adm), g/dL	3.55 [3.10–4.10]	3.65 [3.20–4.10]	3.50 [3.00–4.10]	0.373
ALC (adm), /uL	1247 [695–2079]	1351 [1020–2834]	1219 [684–2059]	0.100
PNI (adm)	42.2 [35.9–49.1]	43.6 [38.6–55.4]	42.0 [35.7–49.1]	0.239
CONUT (adm)	3.0 [1.0–5.0]	2.5 [0.0–3.2]	3.0 [1.0–5.0]	0.102
mNUTRIC ≥ 5	154 (43.4)	14 (43.8)	140 (43.3)	1.000
**Interventions**				
Ventilation, days	10.0 [5.0–16.2]	13.0 [9.0–17.0]	10.0 [5.0–16.0]	0.056
Mechanical ventilation	276 (77.7)	31 (96.9)	245 (75.9)	0.003
ICP monitoring	146 (41.1)	16 (50.0)	130 (40.2)	0.347
CRRT	27 (7.6)	4 (12.5)	23 (7.1)	0.287
Osmotherapy	326 (91.8)	31 (96.9)	295 (91.3)	0.496
Mannitol	244 (68.7)	24 (75.0)	220 (68.1)	0.549
Hypertonic saline	301 (84.8)	27 (84.4)	274 (84.8)	1.000

Rows in bold are variable-group headings. Values are median [IQR] or *n* (%). Mann–Whitney U (continuous) or Fisher exact (categorical) tests. Comorbidities defined by a recorded diagnosis date. ^a^ The *p*-value shown on the first row of the admission diagnosis block (0.033) is an omnibus chi-square test across the seven diagnosis categories, not a test of any single category. Abbreviations: GRV, gastric residual volume; GCS, Glasgow Coma Scale; APACHE II, Acute Physiology and Chronic Health Evaluation II; SOFA, Sequential Organ Failure Assessment; SAH, subarachnoid hemorrhage; ICH, intracerebral hemorrhage; AIS, acute ischemic stroke; TBI, traumatic brain injury; ALC, absolute lymphocyte count; PNI, Prognostic Nutritional Index; CONUT, Controlling Nutritional Status; mNUTRIC, modified Nutrition Risk in Critically Ill; ICP, intracranial pressure; CRRT, continuous renal replacement therapy.

**Table 2:** GRV ≥ 250 and clinical outcomes.

Outcome	GRV ≥ 250, *n* (%)	GRV < 250, *n* (%)	Crude OR (95% CI)	Firth aOR (95% CI)	*p*
In-hospital mortality	6 (18.8)	48 (14.9)	1.39 (0.56–3.46)	1.46 (0.52–3.65)	0.449
ICU mortality	4 (12.5)	20 (6.2)	2.34 (0.79–6.95)	2.26 (0.62–7.03)	0.203
28-day mortality	6 (18.8)	38 (11.8)	1.82 (0.72–4.57)	1.97 (0.69–5.04)	0.194
Poor neurological outcome (GOS 1–3)	23 (71.9)	136 (42.1)	3.40 (1.55–7.45)	4.84 (2.11–12.08)	<0.001

Firth penalized logistic regression adjusted for age, APACHE II, and admission diagnosis. Adjusted models were fitted in the 351 of 355 patients with complete covariates. Crude ORs were computed with the Haldane–Anscombe 0.5-cell correction (Woolf confidence intervals). Crude *p*-values are from Fisher’s exact test. In-hospital mortality is the main endpoint. ICU and 28-day mortality are secondary. Poor neurological outcome (rule-derived GOS category 1–3; Supplementary Table S10) was analyzed as an exploratory outcome. GRV, gastric residual volume; OR, odds ratio; aOR, adjusted odds ratio; CI, confidence interval; GOS, Glasgow Outcome Scale.

**Table 3:** GRV trajectory phenotypes.

Variable	C1 (*n* = 65)	C2 (*n* = 65)	C3 (*n* = 37)	C4 (*n* = 46)	*p*
GRV pattern	Minimal	Low-rising	Early transient	High persistent	
**Demographics**					
Age, years	73 [58–78]	65 [56–72]	68 [57–76]	58 [45–68]	<0.001
Male sex	25 (38)	38 (58)	17 (46)	32 (70)	0.007
APACHE-II	27 [23–31]	28 [26–31]	26 [24–30]	26 [23–29]	0.301
SOFA	4 [2–6]	4 [2–5]	4 [3–6]	4 [2–6]	0.599
Admission GCS	3 [3–4]	3 [3–6]	4 [3–7]	3 [3–6]	0.417
**Laboratory-based nutritional indices—admission**					
mNUTRIC ≥ 5 (high risk)	36 (55)	30 (46)	16 (43)	17 (37)	0.272
Albumin, g/dL	3.50 [3.00–4.00]	3.30 [3.00–3.90]	3.60 [3.30–4.20]	3.80 [3.23–4.20]	0.039
PNI	40.1 [35.0–47.3]	40.8 [35.3–47.6]	41.0 [37.0–46.2]	43.9 [40.2–55.2]	0.024
CONUT score	3.0 [2.0–5.0]	3.0 [1.0–5.0]	3.0 [1.0–5.0]	2.0 [0.0–3.0]	0.018
Malignancy	42 (65)	41 (63)	16 (43)	25 (54)	0.144
**Laboratory-based nutritional indices—ICU discharge**					
Albumin, g/dL	3.20 [3.02–3.50]	3.20 [2.90–3.40]	3.20 [3.02–3.65]	3.30 [3.10–3.52]	0.174
ALC, /µL	1159 [485–1747]	912 [473–1679]	1150 [501–1653]	1444 [881–2298]	0.141
PNI	38.2 [35.0–42.1]	37.3 [33.1–42.7]	37.0 [33.6–43.3]	40.6 [36.4–47.6]	0.028
CONUT score	3.0 [2.0–5.0]	3.5 [2.0–5.0]	3.5 [1.8–5.0]	3.0 [2.0–4.0]	0.069
**GRV trajectory features**					
GRV max, mL	20 [10–60]	70 [35–140]	80 [50–120]	200 [122–298]	<0.001
GRV mean, mL	2 [1–3]	11 [7–19]	11 [5–19]	40 [25–67]	<0.001
GRV measurements, *n*	32 [22–43]	32 [20–49]	37 [28–54]	37 [24–60]	0.301
Ventilation, days	11 [5–14]	9 [6–13]	11 [7–14]	9 [5–17]	0.687
**Intracranial pressure (monitored)**					
ICP monitored	32 (49)	25 (38)	18 (49)	20 (43)	0.610
ICP max, mmHg	24 [15–35]	28 [20–35]	30 [19–42]	31 [25–81]	0.166
ICP > 22 ever	16 (50)	18 (72)	12 (67)	18 (90)	0.026
**Outcomes**					
Poor neurological outcome (GOS 1–3)	30 (46)	23 (35)	16 (43)	21 (46)	0.595
ICU mortality	5 (8)	7 (11)	2 (5)	4 (9)	0.814

Rows in bold are variable-group headings. Values are median [IQR] or *n* (%). Kruskal–Wallis (continuous) or chi-square (categorical) tests across classes. Longitudinal K-means clustering of daily GRV over ICU days 0–14 was performed in patients with at least three measurement days and a first GRV measurement by ICU day 5. C1–C4, trajectory classes; GRV, gastric residual volume; ICP, intracranial pressure; GOS, Glasgow Outcome Scale (rule-derived category; Supplementary Table S10).

## Data Availability

The data presented in this study are available upon reasonable request from the corresponding author. The data are not publicly available due to restrictions imposed by the Institutional Review Board and to protect patient privacy.

## Supplementary Materials

The following supporting information can be downloaded at: https://www.mdpi.com/article/10.3390/nu18183103/s1. Figure S1: Study flow (STROBE). Figure S2: Trajectory curves for K = 3, K = 4, and K = 5. Figure S3: GRV trajectory phenotypes in the primary analysis and in the sensitivity analyses restricted to observed values. Table S1: Baseline comparison of monitored versus non-monitored patients. Table S2: Individual GOS score distribution (1–5) by exposure. Table S3: Sensitivity K-means clustering with K = 3 and K = 5. Table S4: Day-7 landmark analysis for in-hospital mortality. Table S5: GCS motor sensitivity analysis. Table S6: Sensitivity of the early-window GRV–ICP association to opioid-dose specification. Table S7: Sensitivity of the early-window GRV–ICP association to osmotherapy adjustment. Table S8: Sensitivity of the in-hospital mortality estimate to additional confounders. Table S9: Sensitivity analyses of the GRV trajectory phenotypes restricted to observed GRV values. Table S10: Rule used to assign the discharge GOS category and the resulting distribution.
