# Supplementary material for: Gastric Residual Volume as a Candidate Brain–Gut Signal in Neurocritical Care: An Exploratory, Hypothesis-Generating Retrospective Cohort Study
Source: Nutrients. 2026 Sep 21;18(18):3103. doi: 10.3390/nu18183103 (PMC13610725; doi:10.3390/nu18183103)
Supplement: Supplementary file 1 [file nutrients-18-03103-s001.zip › nutrients-4539715-supplementary.pdf]

Supplementary Materials

Gastric Residual Volume as a Candidate Brain–Gut Signal in Neurocritical Care: An Exploratory, Hypothesis-Generating Retrospective Cohort Study

Contents:

Figure S1. Study flow (STROBE).
Table S1. Baseline comparison of monitored versus non-monitored patients.
Table S2. Individual GOS score distribution (1–5) by exposure.
Table S3. Sensitivity K-means clustering with K = 3 and K = 5.
Table S4. Day-7 landmark analysis for in-hospital mortality.
Table S5. GCS-motor sensitivity analysis.
Table S6. Sensitivity of the early-window GRV–ICP association to opioid-dose specification.
Table S7. Sensitivity of the early-window GRV–ICP association to osmotherapy adjustment.
Table S8. Sensitivity of the in-hospital mortality estimate to additional confounders.
Figure S2. Trajectory curves for K = 3, K = 4, and K = 5.
Table S9. Sensitivity analyses of the GRV trajectory phenotypes restricted to observed GRV values.
Figure S3. GRV trajectory phenotypes in the primary analysis and in the sensitivity analyses restricted to observed values.
Table S10. Rule used to assign the discharge GOS category and the resulting distribution.

Figure S1. Study flow (STROBE).

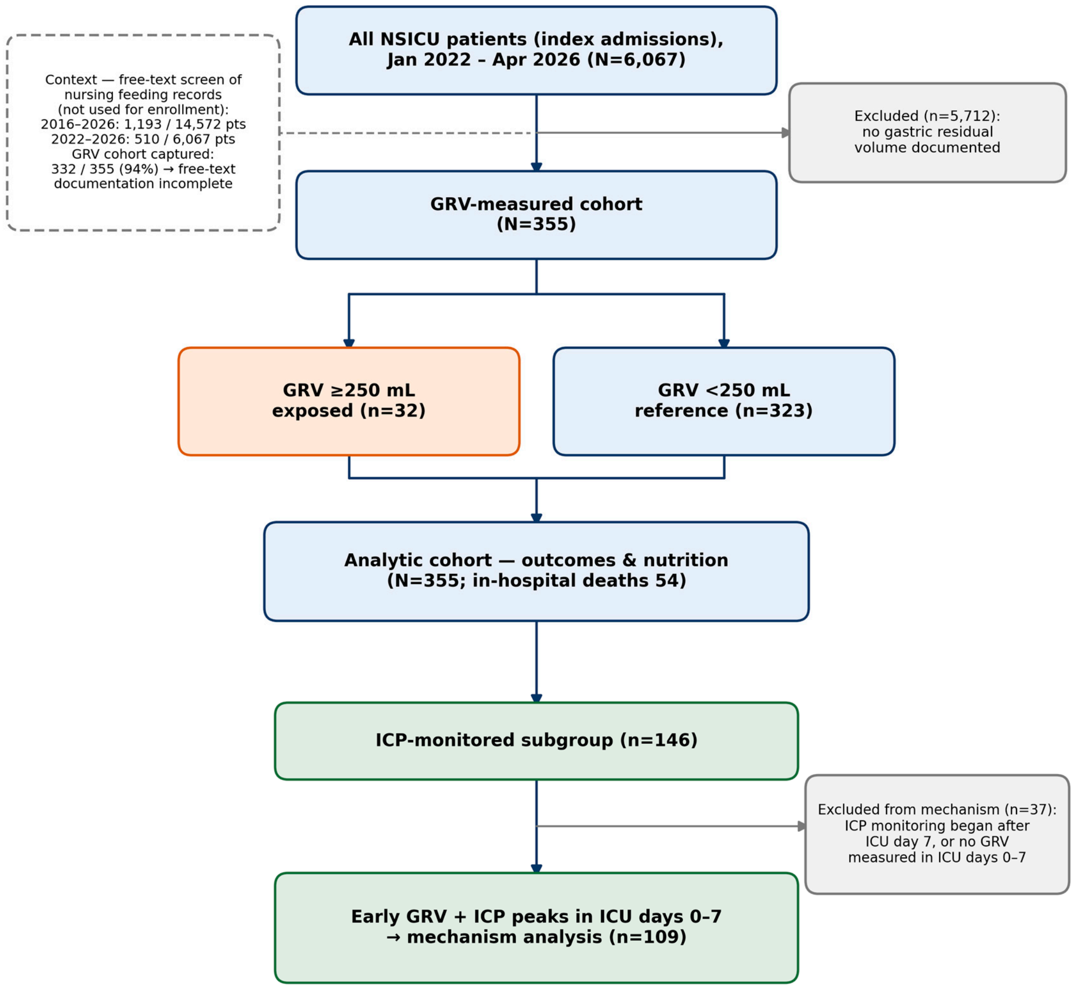

Flow of adult admissions to the Samsung Medical Center neurosurgical intensive care unit (January 2022 – April 2026) through screening for documented gastric residual volume (GRV) measurement, exposure grouping (peak GRV ≥250 mL vs <250 mL), the analytic cohort for the primary in-hospital mortality analysis, and the ICP-monitored mechanism subgroup (n = 146), from which n = 37 patients were excluded because ICP monitoring began after ICU day 7 or no GRV was measured in ICU days 0–7, yielding the early-window mechanism analysis cohort (n = 109). Patients without any documented GRV measurement did not contribute an observed exposure and were not analyzed. The era denominator shown at the top (N = 6,067 patients, index admissions) corrects the value shown in the previous version (14,572), which corresponded to the full 2016–2026 data-warehouse extraction window rather than the study era; the analytic cohort additionally required age ≥18 years. The dashed box provides context from a free-text screen of nursing feeding records: tube-feeding documentation was identified in 1,193 of 14,572 patients (2016–2026) and in 510 of 6,067 patients (2022–2026), yet this screen captured only 332 of the 355 GRV-documented cohort patients (94%); because free-text feeding documentation is incomplete, it was not used for enrollment. STROBE, Strengthening the Reporting of Observational Studies in Epidemiology.

Table S1. Baseline comparison of monitored versus non-monitored patients.

Variable	ICP monitored (n = 146)	Not monitored (n = 209)	p
Age, years	60.0 [51.0–72.0]	67.0 [56.0–76.0]	<0.001
Male sex	69 (47.3)	125 (59.8)	0.023
Admission GCS	3 [3–4]	4 [3–7]	<0.001
APACHE II	27.0 [23.0–30.0]	26.0 [23.0–30.0]	0.491
SOFA	4.0 [1.0–5.0]	4.0 [2.0–6.0]	0.139
Reason for NSICU admission			<0.001
SAH	14 (9.6)	8 (3.8)	
ICH	35 (24.0)	24 (11.5)	
AIS	3 (2.1)	12 (5.7)	
TBI	13 (8.9)	22 (10.5)	
Brain tumor	56 (38.4)	72 (34.4)	
Cerebrovascular, other	6 (4.1)	11 (5.3)	
Other	19 (13.0)	60 (28.7)	
Hypertension	44 (30.1)	75 (35.9)	0.304
Diabetes mellitus	29 (19.9)	68 (32.5)	0.011
Malignancy	84 (57.5)	122 (58.4)	0.913
Chronic kidney disease	7 (4.8)	22 (10.5)	0.075
Chronic liver disease	3 (2.1)	4 (1.9)	1.000
Albumin at admission, g/dL	3.75 [3.12–4.30]	3.40 [3.00–3.92]	<0.001
ALC at admission, /μL	1528 [839–2756]	1090 [605–1758]	<0.001
PNI at admission	44.9 [38.3–53.0]	40.0 [35.3–46.5]	<0.001
CONUT score at admission	2.0 [0.0–4.0]	3.0 [2.0–5.0]	<0.001
mNUTRIC ≥5	58 (39.7)	96 (45.9)	0.277
Mechanical ventilation	129 (88.4)	147 (70.3)	<0.001
Ventilation duration, days	12.0 [6.0–18.0]	9.0 [5.0–15.0]	0.199
CRRT	9 (6.2)	18 (8.6)	0.424
Osmotherapy	143 (97.9)	183 (87.6)	<0.001
Peak GRV, mL	70 [26–150]	45 [10–110]	0.004
Peak GRV ≥250 mL	16 (11.0)	16 (7.7)	0.347
In-hospital mortality	22 (15.1)	32 (15.3)	1.000

Values are median [IQR] or n (%). Mann–Whitney U test (continuous) or Fisher's exact/chi-square test (categorical); the admission-diagnosis distribution was compared by an omnibus chi-square test across categories. Ventilation duration is summarized among ventilated patients. Monitored patients were younger, had lower

admission GCS scores, more often presented with SAH or ICH, more often received mechanical ventilation and osmotherapy, and had more favorable baseline nutritional indices, whereas APACHE II scores and in-hospital mortality did not differ. GCS, Glasgow Coma Scale; APACHE II, Acute Physiology and Chronic Health Evaluation II; SOFA, Sequential Organ Failure Assessment; NSICU, neurosurgical intensive care unit; SAH, subarachnoid hemorrhage; ICH, intracerebral hemorrhage; AIS, acute ischemic stroke; TBI, traumatic brain injury; ALC, absolute lymphocyte count; PNI, Prognostic Nutritional Index; CONUT, Controlling Nutritional Status; mNUTRIC, modified Nutrition Risk in Critically Ill; CRRT, continuous renal replacement therapy; GRV, gastric residual volume; ICP, intracranial pressure.

Table S2. Individual GOS score distribution (1–5) by exposure.

GOS score at hospital discharge	GRV ≥250 mL (n = 32)	GRV <250 mL (n = 323)
1 (death)	6 (18.8)	48 (14.9)
2 (persistent vegetative state)	1 (3.1)	9 (2.8)
3 (severe disability)	16 (50.0)	79 (24.5)
4 (moderate disability)	3 (9.4)	106 (32.8)
5 (good recovery)	6 (18.8)	81 (25.1)

Values are n (%); column percentages may not sum exactly to 100% owing to rounding. Presented descriptively to complement the pre-specified dichotomous analysis (GOS 1–3 versus 4–5): the association with poor neurological outcome was driven primarily by the severe-disability category (GOS 3, i.e., survivors with a discharge GCS of 6–12 or with impaired orientation: 50.0% vs 24.5%) rather than by death (GOS 1: 18.8% vs 14.9%) or the category labelled persistent vegetative state (GOS 2: 3.1% vs 2.8%). GOS, Glasgow Outcome Scale (rule-derived category; Table S10); GCS, Glasgow Coma Scale; GRV, gastric residual volume.

Table S3. Sensitivity K-means clustering with K = 3 and K = 5.

Specification	Class sizes, n	High-GRV class, n	Median peak GRV, mL	Overlap with primary Class 4	ICP >22 mmHg, monitored high class
K = 3	80 / 85 / 48	48	200	45/46 retained (97.8%); purity 45/48 (93.8%)	17/20 (85.0%)
K = 4 (primary)	65 / 65 / 37 / 46	46	200	Reference	18/20 (90.0%)
K = 5	54 / 34 / 58 / 36 / 31	31	230	31/31 from Class 4 (purity 100%); 31/46 retained (67.4%)	14/14 (100%)

Longitudinal K-means clustering repeated with identical preprocessing (log(1+GRV), ICU days 0–14, at least three measurement days, first measurement by day 5), distance metric, initializations, and random seed, varying only the number of classes (K); n = 213. The high-GRV class is the class with the highest class-median peak GRV. “Retained” denotes members of the primary K = 4 high-persistent class (Class 4, n = 46) assigned to the high-GRV class at the alternative K; “purity” denotes the proportion of the alternative high-GRV class originating from the primary Class 4. Intracranial hypertension (ICP >22 mmHg at any time during monitoring) among monitored class members was 54/72/85% across the K = 3 classes, 50/72/67/90% across the K = 4 classes, and 43/72/69/64/100% across the K = 5 classes — highest in the high-GRV class at every K. GRV, gastric residual volume; ICP, intracranial pressure.

Figure S2. Trajectory curves for K = 3, K = 4, and K = 5.

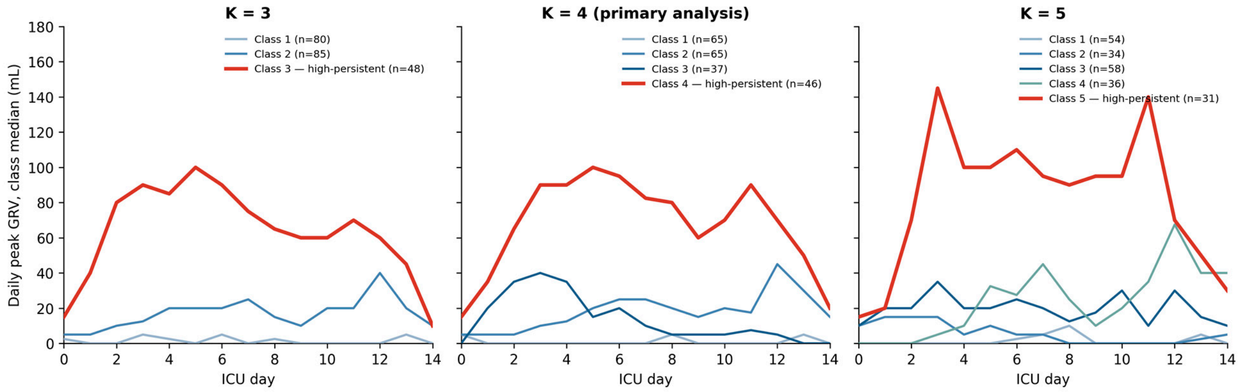

Class-median daily peak GRV (mL) over ICU days 0–14 for each specification of the number of clusters (K); n = 213. Class labels are ordered by class-median peak GRV. A high-persistent phenotype (red) is identified at every K: at K = 3 it retains 45 of the 46 primary Class 4 members, and at K = 5 it condenses to a core of 31 patients, all originating from the primary Class 4. GRV, gastric residual volume.

Table S4. Day-7 landmark analysis for in-hospital mortality.

Analysis	Deaths, exposed	Deaths, unexposed	aOR (95% CI)	p
Day-7 landmark: alive at ICU day 7; exposure = first GRV ≥250 mL within ICU days 0–7	4/20 (20.0)	39/324 (12.0)	2.02 (0.58–5.99)	0.250

Landmark definition: patients whose recorded date of death preceded ICU day 7 were excluded (n = 11; deaths on day 7 itself were retained as events), leaving 344 patients at risk; exposure was a first GRV ≥250 mL within ICU days 0–7 (n = 20), with subsequent in-hospital mortality as the outcome. Firth penalized logistic regression adjusted for age, APACHE II score, and admission diagnosis; 4 patients with missing covariates were excluded (model n = 340). Of the 344 patients at risk, 73 (21%) had no GRV documented within days 0–7 and were necessarily classified as unexposed; in a sensitivity analysis restricted to the 271 patients with at least one GRV measurement in this window, the estimate was similar (aOR 2.57, 95% CI 0.72–7.90, p = 0.136). The landmark estimate is imprecise and consistent in direction with the primary analysis (aOR 1.46); it addresses exposure misclassification arising from whole-stay ascertainment but does not establish the absence of a mortality association or exclude residual time-related bias. aOR, adjusted odds ratio; CI, confidence interval; GRV, gastric residual volume.

Table S5. GCS-motor sensitivity analysis.

Outcome	Model	aOR (95% CI)	p
In-hospital mortality	Primary: age + APACHE II + admission diagnosis	1.46 (0.52–3.65)	0.449
In-hospital mortality	Substituted: age + GCS-motor + admission diagnosis	1.59 (0.57–3.93)	0.357
Poor neurological outcome (GOS 1–3)	Primary: age + APACHE II + admission diagnosis	4.84 (2.11–12.08)	<0.001

Poor neurological outcome (GOS 1–3)	Substituted: age + GCS-motor + admission diagnosis	5.05 (2.20–12.62)	<0.001

Firth penalized logistic regression in the 351 of 355 patients with complete covariates. In the substituted models, the raw GCS motor subscore — which requires no imputation — replaced the APACHE II score as an alternative severity adjustment that does not depend on the verbal component (the APACHE II score itself was derived from the recorded, not the imputed, GCS); because the substitution changes the severity specification as a whole, it does not isolate imputation error alone. Estimates were substantially unchanged. GCS, Glasgow Coma Scale; aOR, adjusted odds ratio; CI, confidence interval; GOS, Glasgow Outcome Scale (rule-derived category; Table S10).

Table S6. Sensitivity of the early-window GRV–ICP association to opioid-dose specification.

**Opioid adjustment**	**Partial Spearman ϱ**	**95% CI**	**p**
None (crude)	0.278	0.095 to 0.443	0.003
Binary opioid — primary specification	0.259	0.073 to 0.427	0.007
MME (fentanyl + hydromorphone + remifentanil)	0.250	0.064 to 0.419	0.009
log(1 + MME)	0.242	0.056 to 0.412	0.012

Values are rank-based partial Spearman correlations between peak GRV and peak ICP over ICU days 0–7 in the ICP-monitored cohort (n = 109). The crude estimate is shown for reference; adjusted estimates account for the specified opioid-exposure term only. p-values use t distributions with $n - k - 2$ degrees of freedom (k = number of conditioning covariates) and CIs the Fisher z transformation. The estimates were similar across opioid specifications. GRV, gastric residual volume; ICP, intracranial pressure; MME, morphine milligram equivalents.

Table S7. Sensitivity of the early-window GRV–ICP association to osmotherapy adjustment.

ICP-monitored cohort, ICU days 0–7 (n = 109). Osmotherapy exposure was near-universal in this window (any 98%, hypertonic saline 92%, mannitol 79%) — a high-exposure collider (treatment of intracranial hypertension); adjustment is therefore reported as sensitivity only.

**Adjustment**	**Partial Spearman ϱ**	**95% CI**	**p**
Crude (unadjusted)	0.278	0.095 to 0.443	0.003
Primary model (opioid, age, diabetes, vasopressor)	0.191	−0.001 to 0.369	0.051
+ Osmotherapy, any (HTS or mannitol)	0.198	0.006 to 0.377	0.044
+ Hypertonic saline	0.197	0.005 to 0.375	0.045
+ Mannitol	0.190	−0.003 to 0.369	0.054

Partial correlations are shown for the primary specification (opioid, age, diabetes, vasopressor) and after additional adjustment for osmotherapy. p-values use t distributions with $n - k - 2$ degrees of freedom and CIs the Fisher z transformation. The magnitude of the association was similar across specifications; the fully adjusted and mannitol-adjusted estimates did not reach statistical significance ($p = 0.051$ and $p = 0.054$, respectively). HTS, hypertonic saline.

Table S8. Sensitivity of the in-hospital mortality estimate to additional confounders.

Model	Firth aOR (95% CI)	P
Primary spec (age+APACHE+dx)	1.46 (0.52-3.65)	0.449
+ Mechanical ventilation	1.26 (0.45-3.16)	0.641
+ Osmotherapy	1.45 (0.52-3.63)	0.457
+ MV + Osmotherapy	1.26 (0.45-3.16)	0.645

Firth penalized logistic regression for in-hospital mortality. The primary estimate is robust to adding mechanical ventilation and/or osmotherapy. aOR, adjusted odds ratio; CI, confidence interval; MV, mechanical ventilation.

Table S9. Sensitivity analyses of the GRV trajectory phenotypes restricted to observed GRV values.

(A) Summary of the primary and sensitivity analyses (n = 213 in each analysis).

Analysis	Data used	Class sizes C1/C2/C3/C4	High-GRV class, n	Primary Class 4 members retained, n/46 (%)	Composition of the high-GRV class	ICP >22 mmHg by class, n/N (%) a	Median peak GRV by class, mL b
Primary: time-series K-means (Euclidean)	Observed values on days 0–14 with interpolation and carry-forward/backward (48% of patient-days imputed)	65/65/37/46	46	46/46 (100)	—	16/32 (50) · 18/25 (72) · 12/18 (67) · 18/20 (90)	20 · 70 · 80 · 200
Sensitivity A: latent class growth analysis (MAR)	Observed values only; no imputation	76/73/44/20	20	20/46 (43.5)	20 from Class 4	17/35 (49) · 23/33 (70) · 12/15 (80) · 12/12 (100)	20 · 70 · 120 · 300
Sensitivity B: time-series K-means (DTW)	Series truncated at the last observed day; interpolation confined to gaps between observations; no carry-forward or carry-backward	61/44/72/36	36	27/46 (58.7)	27 from Class 4, 6 from Class 2, 3 from Class 3	13/27 (48) · 14/22 (64) · 22/30 (73) · 15/16 (94)	20 · 60 · 78 · 175

(B) Cross-tabulation of the primary classes (rows) against the sensitivity-analysis classes (columns).

Primary class	LCGA C1	LCGA C2	LCGA C3	LCGA C4 (high-GRV)		DTW C1	DTW C2	DTW C3	DTW C4 (high-GRV)
Primary C1 (n = 65)	59	5	1	0		53	10	2	0
Primary C2 (n = 65)	7	55	3	0		7	3	49	6
Primary C3 (n = 37)	10	6	21	0		0	27	7	3
Primary C4 (n = 46)	0	7	19	20		1	4	14	27
Total	76	73	44	20		61	44	72	36

(C) Observed daily peak GRV in the high-GRV class of each analysis: class median (mL) and number of patients with an observed value on that day.

**Class (ICU day →)**	**0**	**1**	**2**	**3**	**4**	**5**	**6**	**7**	**8**	**9**	**10**	**11**	**12**	**13**	**14**
Primary Class 4 (n = 46) — median	15	35	65	90	90	100	95	82	80	60	70	90	70	50	20
Primary Class 4 (n = 46) — n observed	7	22	32	36	35	35	31	32	28	26	21	19	17	17	18
LCGA high-GRV class (n = 20) — median	20	20	100	180	152	165	130	130	105	95	95	140	70	50	30
LCGA high-GRV class (n = 20) — n observed	2	7	11	15	14	18	17	17	16	15	14	14	13	13	13
DTW high-GRV class (n = 36) — median	48	68	70	90	50	70	60	60	58	40	65	50	40	65	90
DTW high-GRV class (n = 36) — n observed	2	10	22	28	31	33	30	30	26	24	18	17	16	14	13

GRV, gastric residual volume; ICP, intracranial pressure; LCGA, latent class growth analysis; DTW, dynamic time warping; MAR, missing at random; ICU day, days from the index NSICU admission. a Among monitored patients (ICP >22 mmHg at any time during monitoring); the proportion was highest in the high-GRV class in every analysis but is based on small and overlapping subsets. b Median of each patient's peak GRV over all documented days, as in Table 3 of the main text. Patient-day accounting: the 15-day window comprised 3,195 patient-days, of which 1,657 (52%) had an observed value and 1,538 (48%) were imputed in the primary analysis: 981 (31%) were carried forward after the last observed day, 458 (14%) were carried backward before the first observed day, and 99 (3%) were interpolated between observations. The last observed GRV value defined the end of the observed series and did not necessarily coincide with ICU discharge or death (documentation also ends when enteral feeding is stopped or oral intake resumes); it preceded day 14 in 155 of 213 patients (73%; median last observed day 9, IQR 6–14; median 7 observed days per patient, IQR 5–11). The index NSICU stay recorded in the registry ended before day 15 in 165 patients (78%), but this boundary was not used because readmissions to the NSICU and transfers to other ICUs were not linked in the registry extract. Sensitivity A: finite mixture of class-specific cubic polynomial trajectories of log(1 + GRV) with a common residual variance, estimated by expectation–maximization (60 random starts, convergence tolerance 10^{-6}, all starts converged) using observed values only, under a missing-at-random assumption that cannot be verified; K = 4 was fixed for comparability with the primary analysis (BIC 6,336.9, 6,327.2, and 6,329.9 for K = 3, 4, and 5); classification entropy 0.69, mean posterior probability of assigned class 0.75–0.91 across classes, maximum posterior probability <0.7 in 64 of 213 patients (2 of 20 in the high-GRV class). Sensitivity B: time-series K-means with dynamic-time-warping distance (tslearn 0.9.0; 30 initializations; random seed 42) on variable-length series running from the first to the last observed day (median length 8 days, range 3–15); because ICU episodes were not linked, interpolated gaps between observations could include intervals outside an ICU. In both sensitivity analyses classes were numbered by ascending median peak GRV. The 26 primary Class 4 members not retained in the LCGA high-GRV class had fewer observed days (median 6 vs 11), an earlier last observation (median day 8 vs 14), and lower observed peaks (median 130 vs 300 mL) than the 20 retained members; class size and membership should therefore be regarded as sensitive to missing-data handling and to the modeling approach, and the fitted LCGA trajectory for the high-GRV class after day 10 rests on 13–14 patients still under observation.

Figure S3. GRV trajectory phenotypes in the primary analysis and in the sensitivity analyses restricted to observed values.

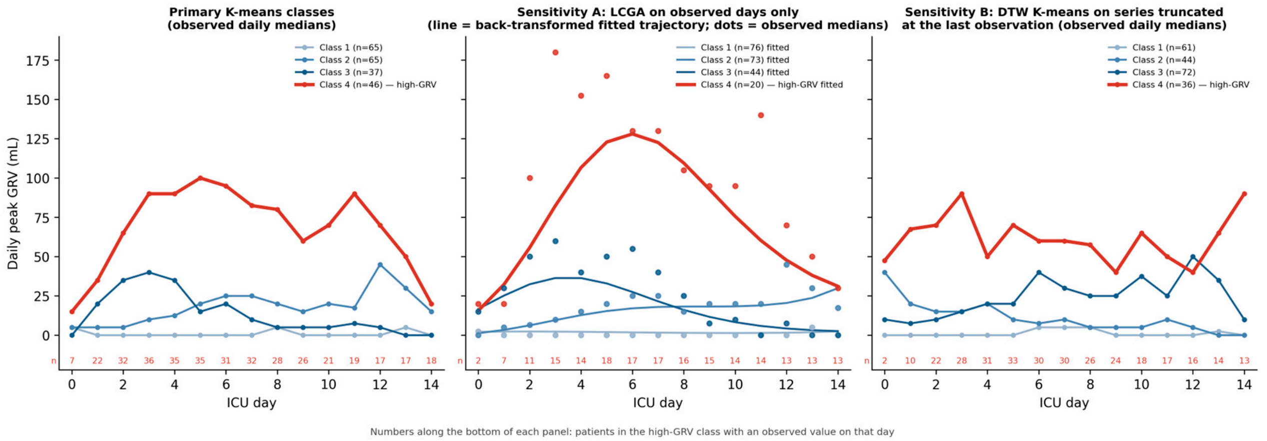

Left: observed daily medians of daily peak GRV for the primary time-series K-means classes. Middle: sensitivity analysis A — latent class growth analysis fitted to observed values only (lines, class trajectories back-transformed from the log(1 + GRV) scale; dots, observed daily medians). Right: sensitivity analysis B — time-series K-means with dynamic-time-warping distance on series truncated at the last observed day (observed daily medians). Classes are numbered by ascending median peak GRV; the high-GRV class of each analysis is shown in red, and the numbers along the bottom of each panel give the patients in that class with an observed value on each day. Details in Table S9.

Table S10. Rule used to assign the discharge GOS category, in the order applied, and the resulting distribution (n = 355).

Step	Condition (records closest to hospital discharge)	Assigned category	n
1	In-hospital death	GOS 1 (death)	54
2	Discharge GCS 3–5	GOS 2 (persistent vegetative state)	9
3	Discharge GCS 6–12	GOS 3 (severe disability)	76
4	Discharge GCS 13–15 with none of the three orientation items correct, or with a level of consciousness of P or U on the AVPU scale	GOS 3 (severe disability)	15
5	Discharge GCS 13–14 not meeting step 4	GOS 4 (moderate disability)	82
6	Discharge GCS 15 with one or two orientation items correct, or with a positive delirium screen (CAM-ICU)	GOS 4 (moderate disability)	7
7	Discharge GCS 15 with all three orientation items correct (n = 20) or with orientation not recorded (n = 67), and no positive delirium screen	GOS 5 (good recovery)	87
8	Discharge GCS not recorded: unresponsive on the AVPU scale (or RASS ≤ −4)	GOS 2	1
9	Discharge GCS not recorded: responsive to voice or to pain only on the AVPU scale, or alert with none of the orientation items correct	GOS 3	4
10	Discharge GCS not recorded: alert on the AVPU scale (GOS 5 if all three orientation items were correct)	GOS 4	20

GOS, Glasgow Outcome Scale; GCS, Glasgow Coma Scale; AVPU, alert/verbal/pain/unresponsive scale; RASS, Richmond Agitation–Sedation Scale; CAM-ICU, Confusion Assessment Method for the ICU. The rule was implemented when the study database was constructed, before the present analyses, and is applied in the order shown (the first condition met determines the category). It uses the last recorded GCS, AVPU level, orientation assessment (three items), and delirium

screening before hospital discharge (median 17 hours before discharge; within 24 hours in 247 of 326 patients with a recorded GCS); when re-applied in this order, it reproduced the stored category in all 355 cohort patients and in all 13,815 database records with an assigned category. For the 136 survivors with an artificial airway at discharge, the verbal component of the discharge GCS was imputed by the Rutledge regression method. The GOS labels denote the numbering of the categories: the rule reflects consciousness and cognitive status at discharge and does not assess functional independence, so the categories should not be read as validated functional outcome classes, and the distinction between moderate disability and good recovery in particular depends on orientation items that were not recorded in 67 patients. The outcome was therefore analyzed only as the binary contrast of GOS 1–3 (poor) versus GOS 4–5 (favorable), for which only the GOS 3/4 boundary (discharge GCS ≤12, or complete disorientation, or a non-alert AVPU level) is decisive; the sensitivity analyses of the airway-related handling are reported in Results 3.3 of the main text.
